# Migratory behaviour and survival of Great Egrets after range expansion in Central Europe

**DOI:** 10.7717/peerj.9002

**Published:** 2020-04-30

**Authors:** Radosław Włodarczyk, Daria Szafara, Krzysztof Kaczmarek, Tomasz Janiszewski, Piotr Minias

**Affiliations:** 1 Department of Biodiversity Studies and Bioeducation, Faculty of Biology and Environmental Protection, University of Łódź, Łódź, Poland; 2 Student’s Ornithological Section, University of Łódź, Łódź, Poland; 3 Department of Electrocardiology, Medical University of Łódź, Łódź, Poland

**Keywords:** Migratory behaviour, Survival, Great Egret, Range expansion

## Abstract

Great Egret *Ardea alba* is one of few Western Palearctic species that underwent a rapid range expansion in the recent decades. Originally breeding in central and eastern Europe, the species has spread in northern (up to the Baltic coast) and western (up to the western France) directions and established viable breeding populations throughout almost entire continent. We monitored one of the first Great Egrets colonies established in Poland to infer migratory patterns and survival rates directly after range expansion. For this purpose, we collected resightings from over 200 Great Egret chicks marked between 2002–2017 in central Poland. Direction of migration was non-random, as birds moved almost exclusively into the western direction. Wintering grounds were located mainly in the western Europe (Germany to France) within 800–950 km from the breeding colony. First-year birds migrated farther than adults. We found some, although relatively weak, support for age-dependent survival of Great Egrets and under the best-fitted capture-recapture model, the estimated annual survival rate of adults was nearly twice higher than for first-year birds (*φ*_*ad*_ = 0.85 ± 0.05 vs. *φ*_*fy*_ = 0.48 ± 0.15). Annual survival rate under the constant model (no age-related variation) was estimated at *φ* = 0.81 ± 0.05. Our results suggest that Great Egrets rapidly adapted to novel ecological and environmental conditions during range expansion. We suggest that high survival rate of birds from central Poland and their western direction of migration may facilitate further colonization processes in western Europe.

## Introduction

Many animal species undergo rapid changes in their biogeographical distribution, which may be driven by a variety of mechanisms (Newton, 1998; Pigot, Owens & Orme, 2010; Bradshaw et al., 2017). Some of these mechanisms act on the population level, resulting from changes in basic demographic parameters such as reproduction, survival or recruitment age (Duncan, Blackburn & Veltman, 1999; Menu, Gauthier & Reed, 2002). Others are related to ecological traits of the species, e.g., dispersal level, behavioural plasticity or tolerance toward human presence (Blondel, Chessel & Frochot, 1988; Devictor, Julliard & Jiguet, 2008; Cornelius et al., 2017). Finally, human activity and its consequences, such as climate change, habitat loss or alterations in agricultural practices, are the major determinants of species distribution (Chamberlain et al., 2000; Melles et al., 2011; Virkkala & Lehikoinen, 2017).

Individuals that colonize novel habitats face wide range of challenges, as they lack knowledge about local food resources, predation or human disturbance level (West-Eberhard, 2003). Insufficient information about local environment can reduce their survival, breeding success or recruitment rate (Duckworth, 2008). Exploration of novel areas is often ephemeral and does not necessarily lead to the establishment of stable populations, but it can also lead to the extension of original distribution range (Sax, Stachowicz & Gaines, 2005; Kokko & López-Sepulcre, 2006). The mechanisms of range expansions are usually complex and case-specific, as they can be driven by a multitude of environmental, ecological and demographic factors (Blackburn, Lockwood & Cassey, 2009; Duckworth & Badyaev, 2007). Great Egret *Ardea alba* provides a noticeable example of recent breeding range expansion among the western Palearctic birds, but the causes of this process and its consequences for newly established populations have received little scientific attention (Ławicki, 2014).

Great Egret is a relatively common species with a worldwide distribution (Del Hoyo, Elliott & Sargatal, 1992) and the global population size estimated at 0.6–2.2 mln individuals (BirdLife International, 2019). In the middle of 20th century its breeding colonies were found in central and eastern part of Europe (Bauer & Glutz von Blotzheim, 1966), mainly in the eastern Ukraine close to Black Sea, and along river Danube in Hungary, Bulgaria and Romania (Hagemeijer & Blair, 1997). Large colonies were also located on Lake Neusiedl in Austria and at Volga river delta in Russia (Bauer & Glutz von Blotzheim, 1966). Significant increase in the European population size and expansion to the west and north of the continent was recorded at the end of 20th century. In consequence, in the 21st century, the Great Egret was listed for the first time as a breeding species in thirteen new European countries (Ławicki, 2014). Currently, breeding populations from Belarus, France, Netherlands, Poland and Latvia are estimated to exceed 100 pairs in each country. The expansion continues and is reflected not only by the growing number of breeding pairs, but also by an establishment of stable wintering populations in the newly colonized parts of the range, even in the harsh climate of northern Europe (Ławicki, 2014).

The aim of the study was to examine migratory patterns and survival of Great Egrets fledged in a breeding colony established after the range expansion in central Poland. The very first breeding attempt of Great Egrets in Poland was documented in 1863, but this was probably an accidental event not followed by the regular presence of breeders (Tomiałojć, 1972) and breeding of the species has not been observed for the next hundred of years. A rapid increase in the number of observations of non-breeding individuals started from the middle of the 20th century, and the second breeding event was recorded in 1997, when three nests were found at Biebrza Marshes (Pugacewicz & Kowalski, 1997). At the beginning of the 21st century, nesting Great Egrets were recorded at eleven locations, but most of these sites were ephemeral and birds did not breed there regularly. By 2010, only two permanent colonies occurred in Poland, one at Biebrza Marshes and the second at the Jeziorsko reservoir (Janiszewski, 2009). We monitored the fates of birds fledged in the latter colony since it has been established in 2002.

## Materials & Methods

The study was performed at Jeziorsko reservoir, central Poland (51°40′N, 18°40′E). Every spring in 2002–2017 all areas suitable for Great Egrets were visited to find the exact location of the breeding colony. In 2010, 2014, and 2016 breeding of Great Egrets on the reservoir was not confirmed, possibly because we did not find the location of the colony. In the remaining years, the colony was visited regularly from May to July in order to ring chicks, but we avoided visits at the early reproductive stages (laying and incubation) to minimize disturbance. In total, we ringed 216 chicks from 82 nests. Each chick was marked with metal and plastic ring; the latter was put on tibia to increase detectability in the field. Bill and tarsus length were measured to estimate age of each chicks. Catching, ringing, and handling birds was performed with permission from the Polish Academy of Sciences, with the approval of the Ministry of Environment in Poland and General Environmental Protection Directorate in Poland (DZP-WG.6401.03.2.2018.jro).

Data on resightings and recoveries were obtained from the database of Polish Ringing Centre. Until 31.12.2019, we collected 110 resightings from 51 individuals (1.5 observation per individual, range: 1–17). We also obtained five ring recoveries from dead birds. Overall, 30% of resightings were collected within one year period from ringing date. Polish Ringing Centre obtains resighting data from a wide range of professional and unprofessional observers, as all the information is collected using a website server open to the public (http://www.stornit.gda.pl). All Great Egrets from our study population were marked with large plastic leg rings that increase probability of resightings by unprofessional birdwatchers and nature photographers, who often visit areas attractive for waterbirds. Thus, our resighting data was largely independent from the activities of professional ringing schemes across Europe and resighting effort should be relatively even across the most of western and central European countries. Taking all this into account, we did not expect any major geographical biases in our data.

We used geographical coordinates of resightings and recoveries to calculate the direction and distance of migration using a loxodromic formula (Imboden & Imboden, 1972), where north was referred to as 0°. All observations were divided into three stages of annual cycle: wintering period (December–February), migrations (March–April for spring migration, and August–November for autumn migration), and breeding season (May–July). For each stage we calculated the mean gravity point expressed as the mean of the coordinates of all observation points, as in other studies (Bairlein, 2001; Remisiewicz, 2002). For individuals observed multiple times at the same location we used only one resighting per month. Also, resightings within a radius of 10 km in one month where treated as a single observation. After this treatment, the dataset consisted of 99 observation points. These data were used to calculate mean ± SD for the angle of migration using circular statistics in Oriana 2.0 (*Kovach Computing Services, Anglesey, Wales*) software. Differences in the angle of migration between successive stages of life cycle were tested using Watson-Williams test (Batschelet, 1981). The mean distance of recoveries of egrets observed in different months was compared by ANOVA and post-hoc Tukey’s test (Zar, 1996).

To analyse survival we used capture-recapture models implemented in Mark software (White & Burnham, 1999). We estimated two population parameters: survival probability (*φ*) and resighting probability (*p*) using Cormack–Jolly–Seber (CJS) models for live recaptures. We grouped all resightings into 15 encounter occasions for each bird (one year duration, 2004–2018), where the first encounter occasion after ringing started from the beginning of the first breeding season (beginning of May). First, we tested goodness-of-fit of our data to the fully time-dependent CJS model using RELEASE test 2 + test 3 approach and we found no evidence for the lack of fit (*χ*^2^ = 5.04, *df* = 15, *p* = 0.99). Second, we fitted constant and time-dependent (between-year variation) models for both parameters (*φ* and *p*) and we also tested for the effects of age (first-year versus adult) and hatching date on survival probability, but the latter effect was fitted only for the first-year birds in the age-dependent model. The Akaike Information Criterion adjusted for small sample size (AIC_C_) was used to compare relative fit of the models and the lowest AIC_C_ value indicated the most parsimonious model. The models were also compared with Akaike weights, which are interpreted as the weights of evidence in favour of a given model against all other fitted models. All values are presented as means +/- SD.

## Results

### Wintering areas and migration

**Figure 1:**
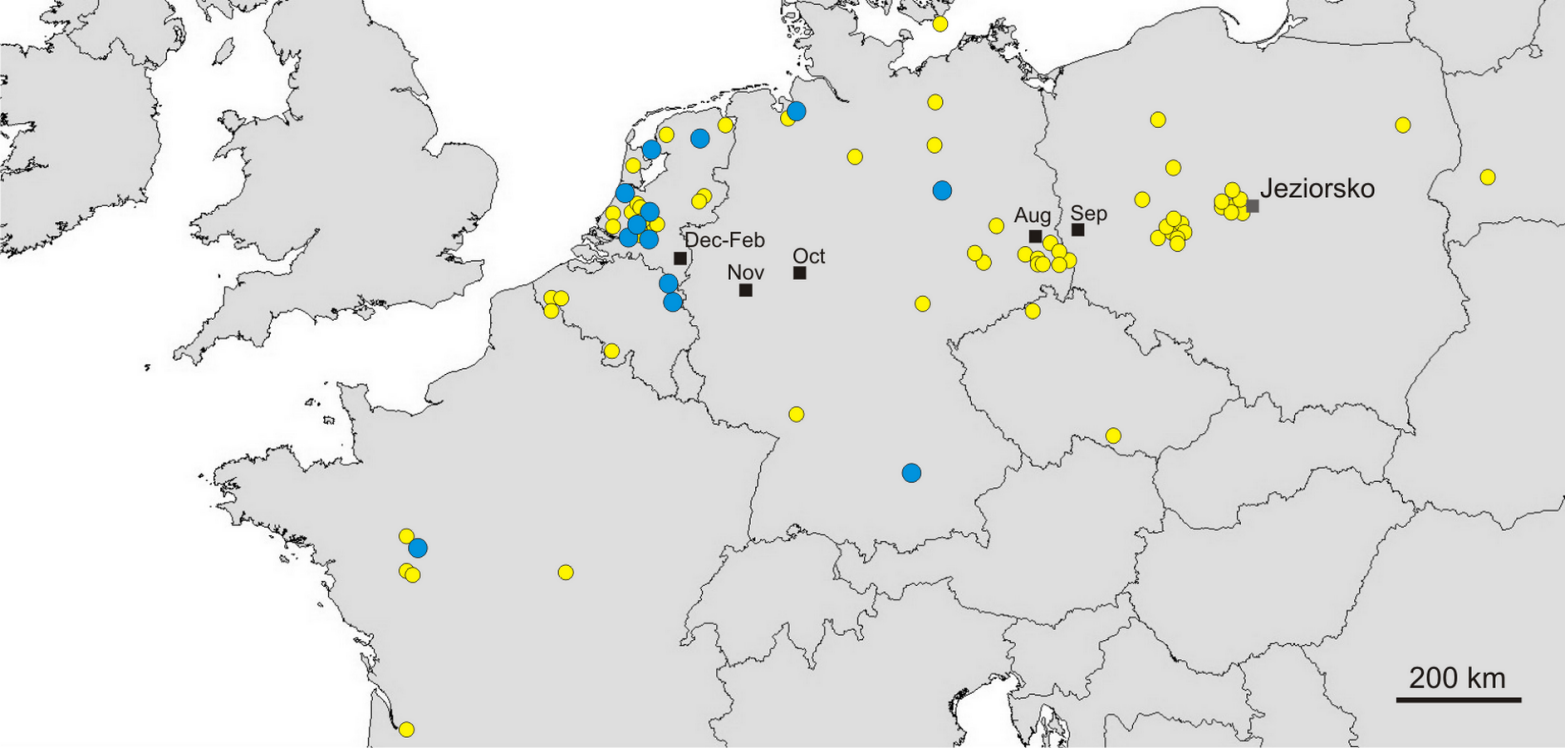
Autumn (yellow circles) and winter (blue circles) resightings of Great Egrets from breeding colony at the Jeziorsko reservoir, central Poland. Mean gravity points of resightings from different periods are marked with black squares. Yellow circles represent autumn (August–November) resightings and blue circles represent winter (December–February) resightings. Mean gravity points of resightings from different periods, indicated by the moths, are marked with black squares.

**Figure 2:**
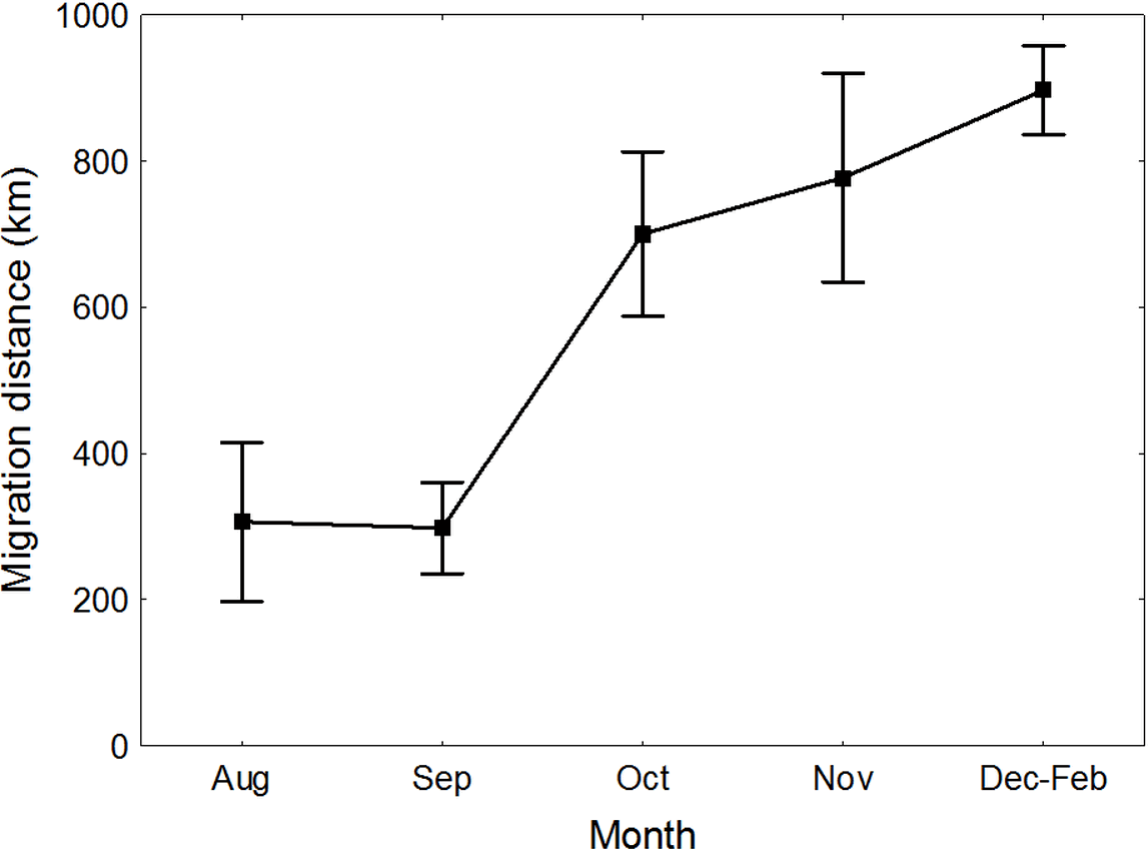
Migration distance of Great Egrets from central Poland during autumn and winter period. Means ± SE are presented.

**Figure 3:**
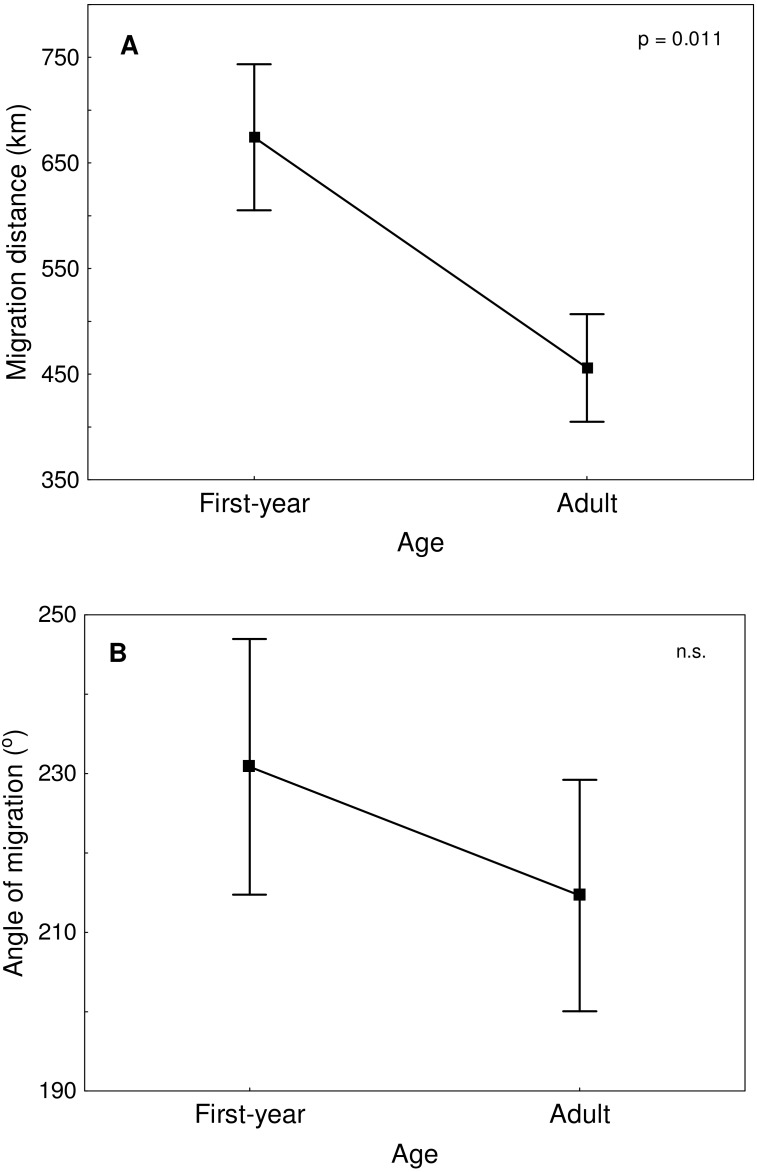
Migration distance (A) and angle of migration (B) of first-year and adult Great Egrets from central Poland. Means ± SE are presented.

Great Egrets from our study colony spent winter mainly in the western Europe (Fig. 1). Wintering locations were scattered through France (11%), Netherlands and Belgium (72%) and northern/central Germany (16%). Egrets migrated mainly in the western direction from their breeding colony, and their angles of migration were not randomly distributed (mean = 268.14° ± 12.1°; *Rayleigh test: Z* = 13.39, *p* < 0.05). The mean distance from the breeding colony to the wintering sites was 883 ± 200 km, and most (71%) winter resightings were from a distance of 800–950 km. The farthest reported wintering location was 1414 km away from the colony, when a first-year bird was observed in western France, in Maine-et-Loire region (Fig. 1). Analysis of migratory behaviour during the autumn period revealed a varying rate of migration in the successive months (*F*_5,81_ = 9.55, *p* < 0.001). In August and September birds stayed in a close proximity to the breeding colony (on average closer than 350 km; Figs. 1 and 2), while the migration distance increased rapidly and significantly in October (Tukey’s test: all *p* < 0.05). In fact, many birds probably reached their wintering sites in October, as the mean migration distance did not increase significantly in the following months (Tukey’s test: all *p* > 0.71; Fig. 2). Migratory distance differed significantly between first-year and adult birds (*F*_1,81_ = 6.78, *p* = 0.011, Fig. 3A), as the adults were observed closer to the breeding colony, irrespectively of the month (*F*_5,76_ = 1.57, *p* = 0.18). The mean angle of migration during the autumn period was 264.63° ± 27.9°, similar as for the angle of migration for wintering resightings (*Watson-Williams test: F* = 0.193, *p* = 0.989), which suggested that birds moved directly from breeding to wintering grounds. In fact, only one foreign resighting was reported east of the breeding colony, which was a third-year bird found dead in July at fish pond complex in Belarus, 440 km from the ringing site. Also, the mean angle of migration during autumn period did not differ between adult and first-year birds (*Watson-Williams test: F* = 0.019, *p* = 0.89, Fig. 3B), which suggested that both age groups migrate in similar direction. Resightings from spring migration period were scarce (*n* = 7) and too few for quantitative analyses. The earliest birds were recorded at the breeding grounds at the end of March, but others stayed at the wintering areas until late April. The marked egrets were not observed in breeding colonies other than the natal one. Six birds that fledged at Jeziorsko were reported from this site as adults, which suggested strong philopatry.

### Survival estimates

To estimate survival rate of Great Egrets, we fitted six capture-recapture models (Table 1). Model selection provided support for age-related variation in survival (Table 1), where annual survival rate of adults was nearly twice higher than the estimate for first-year birds (*φ*_*ad*_ = 0.85 ± 0.05 vs. *φ*_*fy*_ = 0.48 ± 0.15). Resighting probability for both age classes was estimated at *p* = 0.10 ± 0.03. The effect of age was, however, relatively weak, as the model with constant survival rate across age classes had only slightly worse fit (Δ*AIC*_*C*_ = 0.31; Table 1). Annual survival rate under this model was estimated at *φ* = 0.81 ± 0.05, whereas resighting probability was *p* = 0.06 ± 0.01. Both models only slightly differed in their relative importance, as measured with Akaike weights (0.44 vs. 0.37; Table 1). We found support neither for the effect of hatching date on survival of first-year birds nor for inter-annual variation in survival rates (Table 1).

## Discussion

The results of our study provide one of the first descriptions of migratory patterns and survival rates of Great Egrets following their recent range expansion in Europe. Resightings of marked birds from the breeding colony at Jeziorsko reservoir, central Poland, indicate that they choose western direction of migration. During winter most birds were observed in Netherlands and Belgium, but certain proportion of individuals stayed closer to breeding grounds, wintering in central and northern Germany. However, we also recorded examples of long migratory distances exceeding 1,400 km, when birds wintered in western France. A tendency of birds from a newly established Polish population to winter in western Europe is reflected by a recent development of stable wintering population of Great Egrets in this part of the continent (Ławicki, 2014). Interestingly, we did not observe movements into southern and eastern direction, where core breeding and wintering areas of the species are located in Europe (Ławicki, 2014). There are no observations of our birds in Danube river valley or in Ukraine. River Danube valley and its estuary was listed as a breeding site for Great Egrets in 19th century (Bauer & Glutz von Blotzheim, 1966) and even during the period of massive egret persecution in Europe this area held relatively large number of birds. Although paucity of ringing data in colonies from the core European range hampers precise identification of migration patterns in these populations, extensive wintering of great egrets in the Mediterranean region, Balkans, and Turkey suggest that many birds from regular breeding grounds head south for winter. Similar pattern is found in the central European populations of the sister species, the Grey Heron *Ardea cinerea*, where southern or south-western migration direction seems to prevail. For example, birds ringed in Czech Republic spend winter mainly in Hungary, Austria, Switzerland and Italy (Cepák et al., 2008), whereas birds from Poland winter mainly in Mediterranean countries: Spain, France and Italy(Manikowska-Ślepowrońska, Mokwa & Jakubas, 2018). Our sightings of Great Egrets suggest that northward range expansion of this species is associated with serious alteration in migration patterns and location of wintering grounds.

**Table 1:** Model selection for survival (*ϕ*) and resighting probability (*p*) of Great Egrets from breeding colony at Jeziorsko reservoir, central Poland. Model subscripts: (.) –constant, age –age variation (first-year vs. adults), date –hatching date, t –time-dependent (annual variation). Models were ranked by ascending AIC_C_.

Model	No. parameters	AIC_C_	ΔAIC_C_	Akaike weight
*ϕ*(age) *p* (.)	3	332.2	0.00	0.44
*ϕ*(.) *p* (.)	2	332.5	0.31	0.37
*ϕ*(age, date) *p* (.)	4	334.0	1.80	0.18
*ϕ*(.) *p* (t)	15	339.2	6.95	0.01
*ϕ*(t) *p* (.)	15	349.1	16.85	0.00
*ϕ*(t) *p* (t)	28	366.5	34.28	0.00

Interestingly, Great Egrets did not show intensive post-breeding dispersal activity. The mean distance of migration was relatively short during the first months of post-breeding period and long-distance migration started in October. During this month we noticed a rapid decline in the number of resightings collected in close proximity to the breeding colony, while the number of long-distance resightings increased. Probably, warm autumns during last decades in Central Europe allow birds to stay close to the breeding sites and maintain good physical condition for a relatively long time. It may be particularly advantageous for egrets to delay migration until October, when majority of carp fish farms move fish to smaller ponds before winter (Turkowski & Lirski, 2010). This procedure provides an easy access to small fish for many wild birds, mainly gulls and herons. Also, water level at dam reservoirs (including Jeziorsko) usually drops down in this period, producing large areas of shallow waters rich in fry. In consequence, egrets can use rich food resources and do not have to start their autumn migration until frosts reduce their access to attractive foraging sites. We were not able to directly test for the relationship between migration behaviour and carp production process, but locations of autumn egret resightings seem to support this scenario. 44% of observations collected from August–October came from fish farms or dam reservoirs, where our marked birds were observed in the mixed flocks of herons and gulls that often preyed on small fish.

Our best-fitted capture-recapture model provided support for lower survival rate of first-year birds, when compared with adults, but the constant model with no age-related variation in survival had only slightly worse fit and, thus, both models were technically non-distinguishable. While the best-fitted model indicated considerable differences in survival between both age classes (*φ*_ad_ = 0.85 vs. *φ*_fy_ = 0.48) and SDs for the estimates were relatively narrow (see the results section), similar fit of the constant model could be due to low sample size of first-year birds and, consequently, insufficient statistical power to convincingly demonstrate any age-related variation. Survival rate under the constant model was estimated at *φ* = 0.81, so our capture-recapture analysis provided strong and consistent evidence for high survival rates of adult egrets, while the evidence for lower survival of first-year individuals must be treated with caution, taking into consideration limitations of our data. Nevertheless, age-related differences in survival, where juvenile individuals show higher mortality than adults, is a widespread phenomenon in birds (Clark & Martin, 2007; Guillemain et al., 2010), and we suggest it is a likely scenario in our study population. In general, juveniles show weaker competitive ability, low level of predation avoidance or poor foraging efficiency in comparison with more experienced adult individuals (Anders et al., 1997; Calvert, Walde & Taylor, 2009; Robinson et al., 2004; Woodrey, 2000). For example, survival of adult Little Egrets *Egretta garzetta* from breeding colony in Camarque was estimated at 74%, whereas in juvenile birds it varied between 6.5% and 55% in different seasons (Hafner et al., 1998). Survival rate of adult Reddish Egret *Egretta rufescens* was 99% per each month of the breeding period and 94% per non-breeding month, resulting in the annual survival rate of 73% (Koczur, Ballard & Green, 2017). A study of Great Egrets in Florida revealed slightly higher survival rate of first-year birds (ranging from 52 to 66%) than our study (48%) (Sepúlveda et al., 1999). First-year Grey Herons from Great Britain showed survival probability between 25% and 72%, which was primarily dependent on winter severity (North, 1979). In populations subjected to high level of shooting pressure survival rate of first-year birds was 33% in Grey Heron (Scandinavia) and 28.9% in Great Blue Heron *Ardea herodias* (the United States) (Olsson, 1958; Owen, 1959). Adult survival in both populations was higher - 76.3% and 75.5%, respectively. Negative impact of shooting activity at fish farms is probably low in Poland, as Great Egret is protected by law and illegal shooting of this species is probably marginal. Our study indicates that survival of Great Egrets (both immatures and adults) from a newly colonized areas in Central Europe is relatively high and comparable to survival rates of other ardeid species from their core populations. This may suggest that Great Egrets rapidly adapted to novel ecological and environmental conditions and that the populations established after northward range expansion are, at least to certain extent, self-sustainable. This hypothesis is supported by several resightings of birds fledged at Jeziorsko reservoir, which returned to their natal colony to breed. In the future, high survival rate of birds from Central Europe may also help to produce a surplus of new recruits that will participate in the colonization processes of Western Europe.

## Conclusions

As far as we are aware, our study provided the first information on migratory behaviour and survival rates in a newly established Great Egret population in central Europe. We found that main wintering areas of our study population were located in the western Europe, which in combination with relatively high survival rate, can promote further expansion of Great Egrets towards western part of the continent. We believe that our study improves the understanding of ecological mechanisms associated with the processes of range expansion, and we plea for a joint effort in large-scale ecological monitoring of avian populations that expand their range.

##  Supplemental Information

10.7717/peerj.9002/supp-1 Supplemental Information 1 Recovery data from ringed birds

10.7717/peerj.9002/supp-2 Supplemental Information 2 Resighting data from ringed birds

## Additional Information and Declarations

### Field Study Permissions

The following information was supplied relating to field study approvals (i.e., approving body and any reference numbers): Fieldwork was performed under the permission from the Regional Environmental Protection Directorate in Łódź, Poland (DZP-WG.6401.03.2.2018.jro).

### Data Availability

The following information was supplied regarding data availability: The raw recovery data of ringed birds are available in the Supplementary Files.
